# Benveniste’s Experiments Explained by a Non-Conventional Experimenter Effect

**DOI:** 10.3390/medicines5020028

**Published:** 2018-03-29

**Authors:** Francis Beauvais

**Affiliations:** Scientific and Medical Writing, 91 Grande Rue, 92310 Sèvres, France; beauvais@netcourrier.com; Tel.: +33-6-6836-5836

**Keywords:** systems biology, probabilistic modelling, experimenter effect, quantum-like correlations

## Abstract

**Background:** Benveniste’s biology experiments suggested the existence of molecular-like effects without molecules (“memory of water”). In this article, it is proposed that these disputed experiments could have been the consequence of a previously unnoticed and non-conventional experimenter effect. **Methods:** A probabilistic modelling is built in order to describe an elementary laboratory experiment. A biological system is modelled with two possible states (“resting” and “activated”) and exposed to two experimental conditions labelled “control” and “test”, but both are biologically inactive. The modelling takes into account not only the biological system, but also the experimenters. In addition, an outsider standpoint is adopted to describe the experimental situation. **Results:** A classical approach suggests that, after experiment completion, the “control” and “test” labels of biologically-inactive conditions should both be associated with the “resting” state (i.e., no significant relationship between labels and system states). However, if the fluctuations of the biological system are also considered, a quantum-like relationship emerges and connects labels and system states (analogous to a biological “effect” without molecules). **Conclusions:** No hypotheses about water properties or other exotic explanations are needed to describe Benveniste’s experiments, including their unusual features. This modelling could be extended to other experimental situations in biology, medicine, and psychology.

## 1. Introduction

The case of the “memory of water” is a past scientific controversy that generated passionate debates in the 1980s and 1990s. Mainly due to the difficulties for other teams to reproduce the disputed experiments and the absence of a theoretical framework, this hypothesis is now forgotten. Only sociologists of science keep an interest for this controversy, which was a revealing example of the functioning of science [1,2,3,4].

The controversy emerged in 1988 after the publication of an article of Benveniste’s team and other scientists in the journal Nature [5]. The experiments described in the article suggested that water kept information of biologically-active molecules that had been so diluted that no molecule could be present in test samples. Of course, these nonconformist ideas that challenged many well-established facts were received with great skepticism in the scientific community. Moreover, these experiments were considered as an attempt to give scientific support to homeopathy. At this occasion the expression “memory of water” was coined by the lay press. Admittedly, recording information in a fluid such as water is not an idea easy to accept and, by the way, no convincing physical evidence has been reported to support it until now. The dispute between Benveniste and the editor of the journal scrambled the debate [6,7,8,9]. However, the purpose of this article is not to tell again the controversy, the details of which can be found elsewhere with the other chapters of the complete story [10,11,12,13].

Despite having been marginalized after this disturbing episode, Benveniste continued to develop his investigations. Other biological models were used (mainly isolated rodent heart and plasma coagulation) and original procedures were developed in order to confirm the initial ideas [14,15,16,17,18,19]. Using electronic devices, Benveniste’s team reported that the “electromagnetic signature” emitted from molecules in solution could be transferred to samples of water, or even recorded on computer memory before being “played” (“digital biology”). The common point of these different procedures (high dilutions, electronic transmission, digital biology) was the apparent demonstration of a dissociation of the “properties” of biologically-active compounds from their molecular support.

An important point in Benveniste’s results, which extend over twenty years, is that they cannot be explained by trivial artefacts, scientific forgery, or good-faith errors [10]. The different biological systems, the numerous collaborators, the blind experiments, and the clear-cut results of proof-of-concept experiments are apparently arguments in favour of molecular-like effects without molecules (Table 1). Conversely, there are also some arguments that explain why Benveniste did not succeed to convince his peers. Thus, in 2001, a robot analyser built by Benveniste’s team was appraised by a multidisciplinary team of experts mandated by the United States Defense Advance Research Projects Agency (DARPA). This robot automatically performed experiments with plasma coagulation. The “molecular signature” of an anticoagulant recorded on the hard disk of a computer was “transmitted” via an electromagnetic field to water samples added to plasma in order to study the effect of “informed water” on coagulation. The tasks of the robot included the random choice of “controls” and “tests” that remained masked until the experiment was finished. In an article published in 2006, the experts concluded that they observed some effects supporting the concepts of digital biology with this system when members of Benveniste’s team were present; the experts were, however, unable to reproduce the results with the robot after the team left [20]. Even though the authors of the article stated that they did not reproduce the effects of “digital biology”, they suggested that an experimenter effect could explain these results, but that a theoretical framework was lacking.

The mitigated results of DARPA experiments illustrated recurring observations of Benveniste’s team, namely the difficulty to reproduce the experiments by other scientists. Moreover, there was a kind of glass ceiling that prevented definitely proving the existence of a local cause responsible for the observed effects. Indeed, blind experiments (with labels masked with a code) led to paradoxical results according to the experimental design. Thus, in in-house blind experiments, “expected” results were obtained, as was the case in open-label experiments. In contrast, in blind experiments with remote supervisors who did not participate to the experiments and compared the observed system states and the labels under a code, the results were no better than random. In other words, the “activated” state was evenly associated with samples supposed to be “inactive” and “active” [21]. It is important to underscore that an “activated” state was nevertheless observed and, whatever its place, its emergence was unexplained by a classical approach. These mismatches, which were not in favour of a local cause, were successively interpreted by Benveniste’s team as handling errors, contamination of water, electromagnetic interferences, “jumps of activity” from tube to tube, etc. The hypothesis of a role of water as an information carrier was, however, not called into question.

In 2008, I drew attention to a possible non-trivial role of the experimenter in these paradoxical experiments and suggested that the issue with the two designs of blind experiments (local or remote) was one of the few—if not the only—scientific facts of this story and perhaps the key to understanding these curious results [22]. I described these experiments in details in a book [23] (now translated into English [10]), more particularly the experiments that were designed as proofs of concept. Then I tempted to decipher the logic of these experiments in a series of articles [21,24,25,26,27]. The purpose of these articles was also to show that these results were consistent and deserved to be considered from a fresh point of view, even though the price to pay was an abandon of the initial hypothesis (namely, a molecular-like effect without molecules).

In order to be convincing, any modelling of Benveniste’s experiments must account for the following facts:

1. Emergence of an “activated state” of a biological system without local cause;
2. Correlations between “labels” and system states; and
3. Mismatches of outcomes in blind experiments with a remote supervisor.

In this article, a probabilistic model is built and these three conditions are met, thus suggesting that Benveniste’s experiments can be described without attributing any role to water or to another local cause.

## 2. Materials and Methods

### 2.1. Rationale for an Uninvolved Point of View

The originality of the present modelling is the description of the experimental situation from the point of view of an agent who remains uninvolved in the experimental process. The description from this standpoint is justified in this section and the interest of this approach will appear later.

If we measure the length or the mass of an object, we easily accept that the measured value pre-exists to the measurement process and exists independently of any observation. If after assessing the mass of an object we obtain a result equal to 1.26 kg, we consider that we have gained knowledge on a property of the object. The name “property” itself strongly suggests that the measured value is an intrinsic characteristic of the object. In other words, the measured values and the object’s properties can be matched on a one-to-one basis. In this section, we will see that the assessment of a *relationship* between different variables cannot be considered as a property of the system alone.

**Figure 1:**
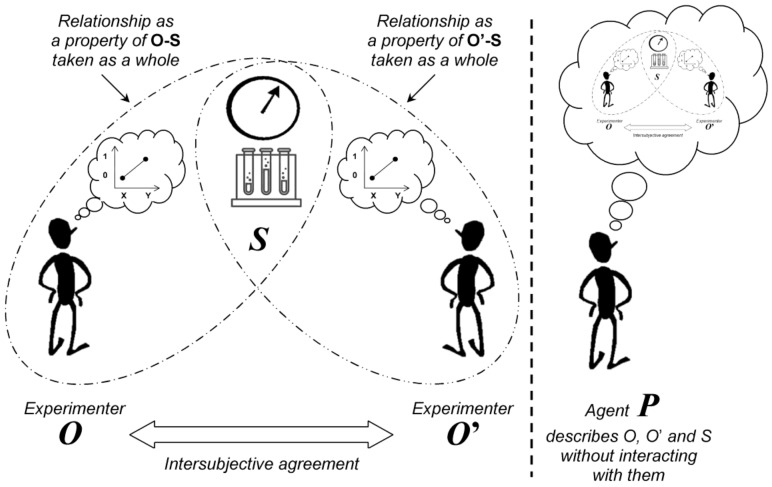
Experimental outcome as a property of system and observer taken as a whole when a relationship is assessed. After a measurement of the experimental system *S* by an experimenter *O* (or *O*’) for a predefined relationship, the measured value is not a property of *S* alone, but is a property of *O*-*S* (or *O*’-*S*) taken as a whole. The experimenters agree on their observations (intersubjective agreement). The situation is described from the standpoint of an agent *P* who does not interact with *O*, *O*’ and *S*. The agent *P* describes the experimental scene (including the experimenters and the observed system) in terms of probabilities of expected outcomes and interactions/measurements. Note that from the point of view of *P*, the order of the interactions of *O*, *O*’ and *S* does not matter: e.g., *O* with *S,* then *O*’ with *S* and, finally, *O-S* with *O*’-*S*; *O* with *S*, and then *O*’ with *O*-*S*.

We suppose an observed system *S* and an experimenter/observer named *O*. The purpose of the experiment is not to measure a single variable of *S*, but to evaluate a relationship between two variables chosen by *O* (e.g., a bet for getting seven with two rolling dices as the first variable and the corresponding outcome as the second variable). The outcome expectation by *O* could be compared to the setting of a measuring device before a measurement. The different possible states of *S* (e.g., the 36 possible outcomes with two rolling dices) are properties that obviously belong to *S*. However, after measurement of *S* for a relationship predefined by *O*, the outcome recorded by *O* (e.g., the observation—or not—of seven with two rolling dices) is not a property of *S* alone, but is a property of *O* and *S taken as a whole* (Figure 1). Another observer, who does not know the predefined relationship, remains ignorant of the specific outcome and he just observes one of the possible outcomes (he does know if *O* won his bet). This demonstrates that the value recorded by *O* is not an obvious property of *S*.

Since *O* and *S* constitute a new “object” *O-S* which cannot be dissociated, one could suggest that a second experimenter would be able to measure it. However, for the same reasons, the consequence of the measurement (i.e., the interaction) of *O-S* by another experimenter *O*’ for the same relationship is the creation of a new entity *O*’-*O-S* that cannot be dissociated (and so on for further observers).

Because the experimental situation cannot be described from an insider point of view—i.e., the perspective of an agent who interacts with *S* and/or *O*—it is described from an outsider point of view. For this purpose, one supposes an agent named *P* who is uninvolved in the measurement process and does not interact with the experimenters when the experiment is performed. This agent describes the experimental scene (including *O*, *O*’ and *S*) in terms of probabilities of expected outcomes and interactions/measurements.

Two spaces are thus defined for the description of the experimental process. The first space is a probabilistic space that is constructed by *P*. This space allows *P* to know on what to expect if he decides to interact with *O-O*’-*S* after the experiment is finished. The second space corresponds to “reality” defined by the intersubjective agreement (*O* and *O*’ always agree on their joint observations/measurements).

### 2.2. Mathematical Description of an Outcome Not Pre-Existing to Measurement

An important consequence of the previous section is that the outcome of a measurement for a relationship *does not pre-exist* to the measurement process. Indeed, if the result is a property of *O-S* taken as a whole and not an individual property of *S*, it means that the result is created when *O* and *S* join together to form *O*-*S*, i.e., when *O* measures *S*.

The second experimenter *O*’ is introduced in the modelling in order to observe the measurement process of *S* by *O* (symmetrically, *O* observes the measurement process of *S* by *O*’) (Figure 1).

We now describe in mathematical terms an outcome that does not pre-exist but is created by the measurement process. We state that, *before the measurement*, the future event expected by *O* (event *A*) and the future event expected by *O*’ (event *B*) are *independent* events in the probabilistic space constructed by *P*. Indeed, suppose that the events *A* and *B* are not independent, but strictly correlated: if the event *B* is defined with certainty (i.e., Prob (*B*) = 0 or 1), then the event *A* is also defined with certainty before being measured. This means that, in this case, the result of the measurement of *S* by *O* pre-exists to this process.

By definition, the two events *A* and *B* are independent if the joint probability of *A* and *B* equals the product of their probabilities:

Prob (*A* ∩ *B*) = Prob (*A*) × Prob (*B*) (1)

The right side of the equation refers to the probabilistic space constructed by the uninvolved agent *P* and the left side refers to the “reality” shared by *O* and *O*’. “Reality” is, thus, defined as the events in the subset *A* ∩ *B* of the probabilistic space constructed by *P*. In other words, each “real” event is an element of the subset *A* ∩ *B* that corresponds to the interaction of *O* and *O*’. The events observed by *O* and *O*’ in the subset *A* ∩ *B* are *coincident events* from the point of view of *P* and, therefore, *do not pre-exist* before the interaction of *O-S* and *O*’-*S* (they are properties of *O*-*O*’-*S* taken as a whole, not properties of *O-S* alone or *O*’-*S* alone).

Combining independence of expected outcomes and intersubjective agreement will be the basis for the construction of a model that describes outcomes not pre-existing to their measurement.

### 2.3. Definitions of “Direct” and “Reverse” Relationships

In most experiments in medicine or biology, the experimenters seek to evaluate a relationship between a “cause” (independent variable) and an “effect” (dependent variable). Control samples in experimental biology (or placebos in clinical trials) allow assessing the effects of variables other than the independent variable, but not controlled by the experimenter.

We propose to describe an elementary experiment aimed at evaluating a relationship between some experimental situations and the corresponding states of a biological system. For simplicity, we suppose that the biological system has only two mutually exclusive states symbolized with “↓” (= “resting” state; not different from background noise) and “↑” (= “activated” state; significantly different from background noise).

We suppose also that the experimental system can be exposed to two experimental conditions that are both control conditions or “placebos”. Their only difference is their “labels” noted *Pcb*_0_ and *Pcb*_1_. Note that labels must be understood in a broad sense; it could be names, colours, procedures, or “rituals”. The term “placebo” is usually reserved in clinical trials when patients think they receive an active treatment although they receive inert pills. In the present case it plays a comparable role, but for the experimenters. Indeed, the experimenters think they manipulate biologically-active samples or procedures but in fact they manipulate only “labels” (all samples and procedures are biologically inert).

A classical approach suggests that the two control conditions are always associated with the “resting” state (i.e., no relationship). This can be translated in mathematical language:

Prob (↓|*Pcb*_0_) = Prob (↓|*Pcb*_1_) = 1 (2)

with Prob (*x*|*y*) which is the conditional probability of *x* given *y* (or the probability of *x* under the condition *y*).

**Figure 2:**
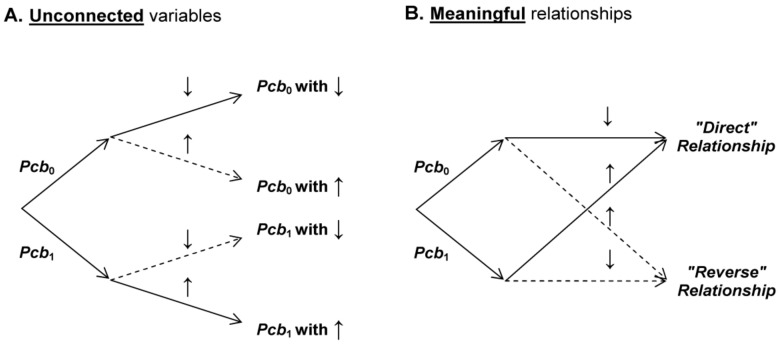
Unconnected variables vs. meaningful relationships. The two experimental conditions named *Pcb*_0_ and *Pcb*_1_ and the two system states (↓, “resting” state; ↑, “activated” state) are described either as unconnected variables (**A**) or as relationships (“direct” or “reverse”) meaningful for the experimenters (**B**).

With two labels (independent variable) and two system states (dependent variable), two *mutually exclusive* relationships can be built (Figure 2). These two relationships are “meaningful” for the experimenters because they result from an association of variables—previously unconnected—that suggest a causal relationship:

1. “Direct” relationship is the association of *Pcb*_0_ with “↓” or *Pcb*_1_ with “↑”; and
2. “Reverse” relationship is the association of *Pcb*_0_ with “↑” or *Pcb*_1_ with “↓”.

The total probability of these two relationships is equal to one and is noted: Prob (*direct*) + Prob (*reverse*) = 1.

The labels *Pcb*_0_ and *Pcb*_1_ play a symmetrical role and consequently Prob (*Pcb*_0_) = Prob (*Pcb*_1_) in probabilistic calculations. Thus, in Figure 2B, according to Equation (2) (no relationship between labels and system states), Prob (*direct*) = Prob (*reverse*) = 1/2. In the present modelling we will explore if, in some conditions, Prob (*direct*) could be different from 1/2.

Note that the definition of direct/reverse relationships is general and does not prejudge which relationship is assessed (e.g., a bet for getting seven with two rolling dices, a double six, etc.). Only the achievement—or not—of the initial objective is assessed.

## 3. Results

### 3.1. Probabilistic Observer-Centred Modelling

For the model of an elementary experiment, we note the probabilities of direct and reverse relationships as Prob (*direct*) = *p* and Prob (*reverse*) = *q*, respectively (with *p* + *q* = 1).

**Figure 3:**
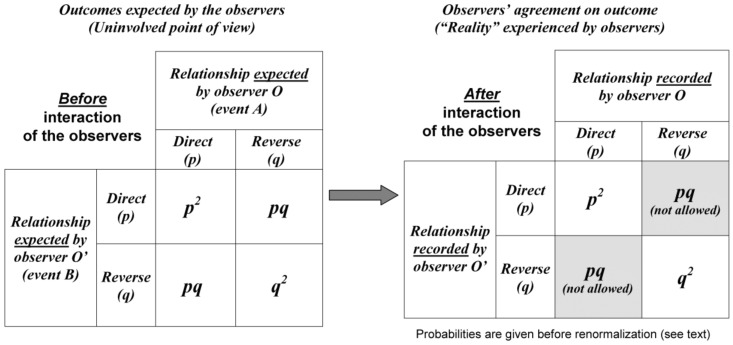
The two spaces of the modelling. The left panel describes the relationship expected by the observers *O* and *O*’ in the probabilistic space described by the uninvolved agent *P*. The two events *A* and *B* are independent because they do not pre-exist to the measurement process (see text). The right panel describes the “reality” experienced by *O* and *O*’ defined by intersubjective agreement (*O* and *O*’ agree on their records). Some situations are not possible (grey areas) and renormalization of probabilities is necessary (see text).

Since the experimental situation is described from the point of view of the uninvolved agent *P*, Prob (*A*) = *p* and Prob (*B*) = *p* in Equation (1). Therefore, *before* the experimenters interact, the probability of a direct relationship is Prob (*direct*) = *p × p* = *p*^2^ and, similarly, Prob (*reverse*) = *q × q* = *q*^2^ according to Equation (1) (Figure 3). *After* the experimenters interact, some situations such as *O* records a direct relationship whereas *O*’ records a reverse relationship are prohibited by intersubjective agreement.

Since the situations that do not fit intersubjective agreement when the observers interact (grey areas in Figure 3) are ruled out, renormalization of probabilities is necessary in order to keep total probability equal to one. The probability of a direct relationship (*p*^2^) is divided by the sum of the probabilities of all possible outcomes (namely, *p*^2^ + *q*^2^):

(3) $$\mathrm{Prob}\;(direct)=\frac{p^{2}}{p^{2}+q^{2}}$$

The numerator and the denominator are divided by *p*^2^ in order to get an equation with only *p* as a variable (taking into account that *p* + *q* = 1):

(4) $$\mathrm{Prob}\;(direct)=\frac{1}{1+{\left(\frac{1}{p}-1\right)}^{2}}$$

This equation is easily generalized to *N* experimenters:

(5) $$\mathrm{Prob}\;(direct)=\frac{1}{1+{\left(\frac{1}{p}-1\right)}^{N}}$$

A particular case of Equation (5) is the absence of experimenters (*N* = 0) that gives Prob (*direct*) = 1/2. After introduction of the experimenters in the modelling, the uninvolved agent *P* replaces *p* with 1/2 in Equation (5) and he calculates that Prob (*direct*) = 1/2.

Remember that with the classical approach, which does not consider experimenters and their outcome expectations, Prob (*direct*) = 1/2. Therefore, the classical approach (the outcome pre-exists to measurement) and the model (the outcome does not pre-exist) lead to the same conclusion. This is consistent with common sense: two “placebos” (or two “controls) are associated with the same “effect” (not different from background noise), regardless of their observation.

At this stage, considering that the outcome pre-exists or not to the measurement process is a matter of personal taste since the same results are obtained in both cases. Nevertheless, in the next section, the consideration of random fluctuations will differentiate these two approaches.

### 3.2. Probabilistic Observer-Centred Modelling with Fluctuations

Random fluctuations are inherent to any measurement or interaction. For the model, we note ±*ε_n_*, a tiny random fluctuation of Prob (*direct*) at time *t_n_* as a positive or negative real number (with |± *ε_n_*| << 1).

Before the observation of the system (*N* = 0), Prob (*direct*) = *p*_0_ = 1/2. At time *t*_1_, the fluctuation of the probability is equal to *ε*_1_. Therefore, *p*_1_ is calculated for *p*_0_ ± *ε*_1_ using Equation (4).

Until now no specific conditions were imposed to the experimental system. However, for the calculation of *p*_2_, there are two possibilities. In the first case, the system comes back to its previous position after each *ε_n_*; in the second case, each state *n* is the starting point for the state *n* + 1. Therefore, we write Equations (6) and (7) that generalize Equation (4) according to these two situations, respectively.

For the first case where the system comes back to its initial position after each fluctuation, *p*_n+1_ is calculated with *p_n_* = *p*_0_ = 1/2:

(6) $${\mathrm{Prob}}_{n+1}\;(direct)=p_{n+1}=\frac{1}{1+{\left(\frac{1}{1/2\pm \varepsilon_{n+1}}-1\right)}^{2}}\;\left(\mathrm{with}\;p_{0}=\;1/2\right)\;=1/2\pm \varepsilon_{n+1}$$

For the second case, each state *n* is the starting point of the state *n* + 1; therefore, each *p_n_* is reintroduced for the calculation of the corresponding *p_n_*_+1_ in a mathematical sequence:

(7) $${\mathrm{Prob}}_{n+1}\;(direct)=p_{n+1}=\frac{1}{1+{\left(\frac{1}{p_{n}\pm \varepsilon_{n+1}}-1\right)}^{2}}\;\left(\mathrm{with}\;p_{0}=\;1/2\right)$$

Equations (6) and (7) refer to experimental systems with different behaviours when submitted to small random fluctuations:

- In the first case (Equation (6)), the experimental system has a structure that is “rigid”. When the system moves slightly apart from its initial position because of a fluctuation (due to thermal agitation, for example), it quickly comes back (the system is repeatedly “set to zero”). In other words, the system “vibrates” around a fixed position and the *mean values* of outcomes are not affected by these tiny vibrations. As examples of such systems, one could cite roulette, coin toss, dice rolling, or a beam splitter that randomly transmits or reflects a photon. Thus, if we put a glass on a table, the probability that it will move a few centimetres from its initial position under the sole action of molecular agitation in a reasonable time lapse can be considered equal to zero in practice. With Equation (6), *p_n_*_+1_ = 1/2 ± *ε_n_*_+1_ (with *p*_0_ = 1/2). This means that with “rigid” systems, despite small fluctuations, Prob (*direct*) remains centred on 1/2 and no relationship is established between “labels” and system states.
- In the second case (Equation (7)), the experimental system may deviate from its initial state after a series of random fluctuations. Each new state of the system after an elementary fluctuation is dependent on the previous one (the successive states are autocorrelated). Thus, a pollen grain at the surface of water will deviate from its initial position to a distant position after a defined time (regardless of the direction) because the grain is sufficiently small to be submitted to the agitation of water molecules. Biological systems, although more complex, are also a good example of such systems. This does not mean that all biological systems are suitable, but some of them can deviate from an initial position (“resting” state) to another position (“activated” state) after a series of random fluctuations. Indeed, biological systems have a “deformable” structure thanks to the rather weak cohesion of their components; the structure of biological systems is intermediary between liquid state (maximal disorder; no structure) and solid state (minimal disorder; structure completely “rigid”).

**Figure 4:**
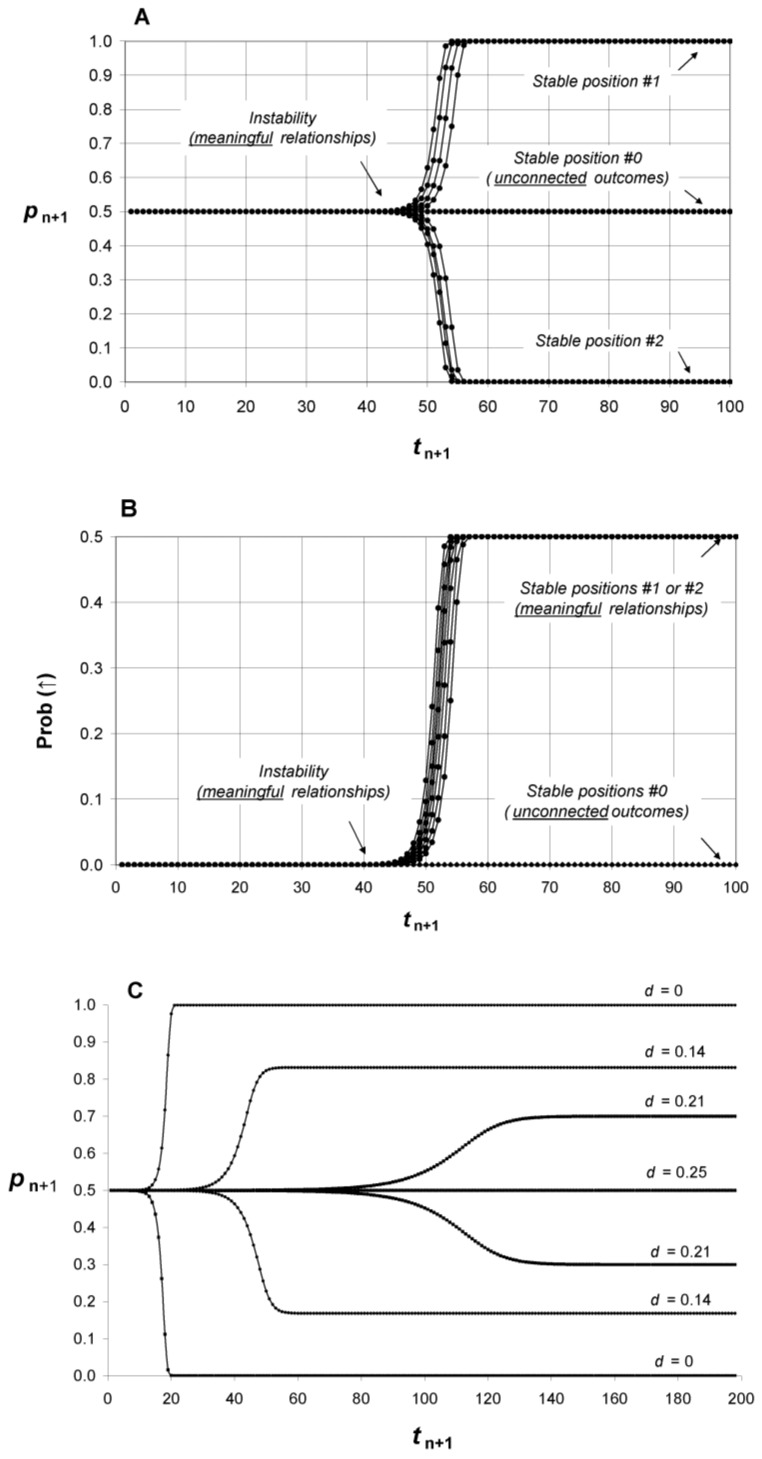
Emergence of relationships between “labels” and states of a deformable system (e.g., a biological system). Panel (**A**) describes the probability to observe a meaningful relationship after *O-S* and *O*’-*S* interact if the fluctuations of the system are taken into consideration (Equation (7)). There is a dramatic transition of the probability from 1/2 toward 1 or 0. If the results expected by the experimenters are the simple sum of unconnected variables then no relationship emerges and Prob (*direct*) remains equal to 1/2. Panel (**B**) corresponds to the emergence of an “activated” state for meaningful relationships. Panel C is obtained by varying the independence of the events expected by *O* and *O*’ (from *d* = 0.25 to *d* = 0) using Equation (15) (see text). The mathematical sequences presented in panels A and B have been obtained after eight computer calculations. Each probability *p*_n+1_ of the sequence is calculated by using *p*_n_ and a probability fluctuations *ε*_n+1_ which is randomly obtained between –0.5 and +0.5 × 10^–15^. For panel (**C**), the range of probability fluctuation was from –0.5 to +0.5 × 10^–5^ for a better display. Note that these figures are obtained before considering that only one of the two stable positions—namely stable position #1—is allowed (see text).

The consideration of tiny probability fluctuations of a “deformable” system as described by Equation (7) introduces *instability* for Prob (*direct*). Indeed, as depicted in Figure 4A, computer simulations show a systematic dramatic transition of Prob (*direct*) from 1/2 toward one of two stable positions. In the stable position #1 where Prob (*direct*) = 1, the relationship between labels and system states is “direct” whereas with stable position #2 where Prob (*direct*) = 0, the relationship is “reverse”. The choice among stable position #1 or #2 is random. In both cases a relationship (direct or reverse) is established between labels and system states.

In fact, only one of the two stable positions is allowed in the probabilistic space constructed by *P*. Indeed, an experiment does not begin when the different measurements are performed, but when the experimental system is prepared. Biological systems are always *prepared in a resting state* (↓) before testing and this state is, therefore, implicitly *labelled as* “*control*”. If the experimenters consider, for example, that samples with label *Pcb*_0_ are for “control” conditions and those with label *Pcb*_1_ are for “test”, then only the stable position #1 is allowed. Otherwise, in the stable position #2, *Pcb*_0_ would be associated to the “resting” state before testing and to the “activated” state after testing.

### 3.3. Consequences of Blind Experiments

When a resting state is achieved, this means that the events *A* and *B* are strictly correlated. For the experimenters *O* and *O*’, it is as if there was a significant causal relationship between labels and system states. In this section, we show that the causal link is only apparent. In addition, we show that a specific blind design offers a possibility to test the model.

Blind experiments are performed in order to avoid classical biases related to the experimenter. We suppose first a supervisor who is a member of the interacting team of experimenters (local supervision). His role is to transmit experimental samples under another name (not meaningful to the experimenters). Note that this task can be also performed by an automatic device. From the point of view of the uninvolved agent *P*, this local supervision is comparable to an open-label experiment (as described in the previous section). Indeed, the assessment of “success” (direct relationship) is performed in all cases locally by a member of the interacting team.

Blind experiments can be also performed with a centralized supervision as frequently done in clinical trials (generally with a statistician supervisor). In this case, the outcomes of the experiments are performed in blind conditions by the experimenters’ team and the results are transmitted to the supervisor who does not assist to the experiments. This remote supervisor assesses the rate of “success” by comparing the list of labels (unknown to the experimenters) and the outcomes (states of the system). In this situation, Prob (*direct*) = Prob (*reverse*) since the assessment of “success” (direct relationship) is not performed locally by a member of the interacting team. Consequently, Prob (*direct*) = 1/2 since Prob (*direct*) + Prob (*reverse*) = 1.

Therefore, blind experiments with different designs offer the possibility to test the model since there are two possible results according to the design of the blind experiment:

1. With local supervisor, Prob (*direct*) = 1 (significant relationship); and
2. With remote supervisor, Prob (*direct*) = 1/2 (no significant relationship).

These results emphasize that the relationship between labels and system states is not causal. Indeed, if there were the case, local and remote assessments of the relationship in blind experiments should lead to the same conclusion. The repetitive failure of the experiments with a remote supervisor was a stumbling block that prevented Benveniste’s team to convince other scientists on the reality of “memory of water”. Therefore, the fact that the difference between local and remote supervision emerges simply from the model is an important result.

### 3.4. Quantum-Like Structure of the Emerging Relationships

The modelling uses only classic probability. Nevertheless, as explained in this section, the logic of the relationship between labels and system states is structured by an underlying quantum-like structure.

According to the law of total probability, the sum of the probabilities of the four outcomes described in Figure 2 is equal to 1:

Prob (*Pcb*_0_) × Prob (↓) + Prob (*Pcb*_0_) × Prob (↑) + Prob (*Pcb*_1_) × Prob (↓) + Prob (*Pcb*_1_) × Prob (↑) = 1 (8)

When the stable position #1 is achieved, Prob (*Pcb*_0_) = Prob (↓) and Prob (*Pcb*_1_) = Prob (↑); for stable position #2, Prob (*Pcb*_0_) = Prob (↑) and Prob (*Pcb*_1_) = Prob (↓). In both cases, by replacing these equalities in Equation (8), we get the same equation:

(9) $${\left[\mathrm{Prob}\;(Pcb_{0})\right]}^{2}+{\left[\mathrm{Prob}\;(Pcb_{1})\right]}^{2}+2\times \mathrm{Prob}\;(Pcb_{0})\times \mathrm{Prob}\;(Pcb_{1})=1$$

We recognize a remarkable identity:

(10) $${\left[\mathrm{Prob}\;(Pcb_{0})+\mathrm{Prob}\;(Pcb_{1})\right]}^{2}=1$$

We introduce now the real numbers *a* and *b* that are defined as Prob (*Pcb*_0_) = *a*^2^ (or *a.a*) and Prob (*Pcb*_1_) = *b*^2^ (or *b.b*). These definitions are for the stable position #1 (note that for the stable position #2, *b*^2^ must be taken equal to −*b ×* −*b*). Equations (2) and (10) are rewritten with *a* and *b*:

(11) $${\left(a\cdot a+b\cdot b\right)}^{2}={\left(a\cdot a\right)}^{2}+{\left(b\cdot b\right)}^{2}+2\times {\left(a\cdot b\right)}^{2}=1$$

Since *a* and *b* are real numbers, (*b.a* − *a.b*)^2^ is equal to zero and can be introduced for symmetry reasons in the equation; moreover (*a.b*)^2^ = (*b.a*)^2^:

(12) $${\left(a\cdot a+b\cdot b\right)}^{2}+{\left(b\cdot a-a\cdot b\right)}^{2}={\left(a\cdot a\right)}^{2}+{\left(b\cdot b\right)}^{2}+{\left(b\cdot a\right)}^{2}+{\left(a\cdot b\right)}^{2}=1$$

1   +  0    =   1/2    +    1/2   = 1(13)

**Figure 5:**
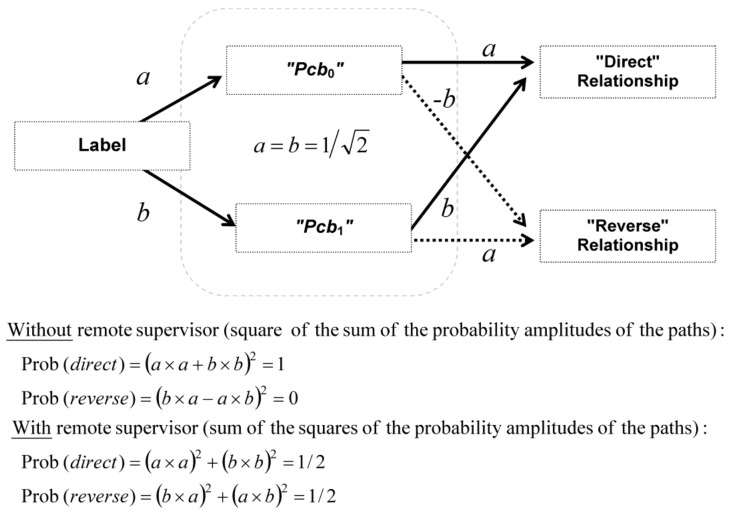
Quantum-like logic of the emerging relationship. The underlying logic of the relationship that emerges in the model is isomorphic to Young’s two-slit experiment. In Young’s two-slit experiment, the screen interferences disappear if the paths of photons are detected. In the model, the relationship between labels and system states is no better than random (i.e., equal to 1/2) if the relationship of labels with system states is assessed by a remote supervisor.

Equation (12) is sketched in Figure 5 for a better understanding. Thus, the left-hand side of Equation (12) is the sum of Prob (*direct*) plus Prob (*reverse*) without a remote supervisor, whereas the right-hand side is the sum of Prob (*direct*) plus Prob (*reverse*) with a remote supervisor. The terms *a* and *b* can be considered as probability amplitudes (their squaring give the corresponding probabilities).

We can recognize in Equation (12) and Figure 5 a mathematical structure that is analogous to single-photon self-interferences in Young’s double-slit experiment (or in a Mach-Zehnder interferometer). In this experiment, photons behave either as particles or waves according to path detection or not, respectively. Path detection is analogous to supervision by a remote supervisor and no path detection is analogous to the absence of supervision by a remote supervisor.

### 3.5. Shift from Unconnected Variables to a Meaningful Relationship

In this section we will deepen the role of the experimenters by studying the progressive shift from a property that belongs only to the system *S* to a property that belongs to *O-S* taken as a whole. For this purpose, we vary the degree of independence of outcome expectations. Equation (1) is generalized by adding the parameter *d*:

Prob (*A* ∩ *B*) = Prob (*A*) × Prob (*B*) + *d* (with 0 ≤ *d* ≤ 1) (14)

**Figure 6:**
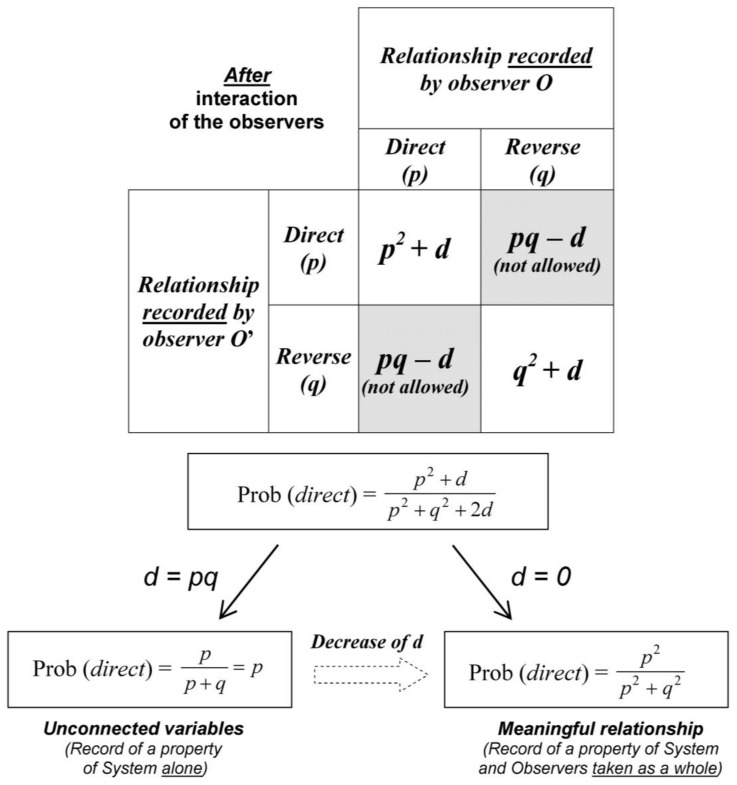
From perfect correlation to complete independence of observers’ expectations. The modelling is generalized in order to consider in the same equation either the record of a property of *S* alone or the record of a property of *O*-*S* and *O*’-*S* (taken as a whole). Thus, the variation of the parameter *d* from 0 to *pq* allows a progressive shift from unconnected variables to a meaningful relationship.

When *d* = 0, the events *A* and *B* are independent and when d increases, their degree of correlation increases. Equation (3) is easily generalized (Figure 6):

(15) $$\mathrm{Prob}\;(direct)=\frac{p^{2}+d}{p^{2}+q^{2}+2d}\;\left(\mathrm{with}\;0\;\leq d\leq pq\right)$$

We have seen that *d* = 0 in Equation (10) and introduction of probability fluctuation leads Prob (*direct*) to a dramatic shift from 1/2 toward 1 or 0. In contrast, with *d* = *pq*, the degree of correlation of the two events *A* and *B* is maximal:

(16) $$\mathrm{Prob}\;(direct)=\frac{p^{2}+pq}{p^{2}+q^{2}+2pq}=\frac{p\times (p+q)}{{\left(p+q\right)}^{2}}=\frac{p}{p+q}=p$$

Probability fluctuations are then introduced in Equation (16):

(17) $$p_{n+1}=p_{n}\pm \varepsilon_{n+1}\;\left(\mathrm{with}\;p_{0}=\;1/2\right)$$

Consequently, if initially *d* = *p_0_q_0_* = 1/4, there is no instability of Prob (*direct*) and Prob (*direct*) fluctuates slightly around 1/2; there is no dramatic transition toward 0 or 1 and no emergence of the “activated” state of *S* (Figure 4A,B). It is as if the outcome *pre-existed* to its measurement since the events *A* and *B* are perfectly correlated. When *d* = *pq*, we see with the help of Figure 1 that *p* is equivalent to the sum of the probabilities of the sub-events considered individually:

*p* = Prob (*Pcb*_0_) × Prob (↓|*Pcb*_0_) + Prob (*Pcb*_1_) × Prob (↑|*Pcb*_1_) (18)

Therefore, varying the value of *d* from *pq* to 0 allows a shift from unconnected variables to a relationship meaningful for the experimenters (Figure 1 and Figure 4C).

In Figure 6, the parameter *d* (from Equation (15)) is an assessment of the *degree of meaning* of the relationship. A low value of *d* (significant meaningful relationship) is obtained after a progressive detachment from the real experimental outcomes toward an abstract entity; during this process the experimental outcomes *lose their identity*. In other words, a “form” is extracted from a cloud of separate dots (like a mathematical curve fitting obtained from experimental outcomes). This abstraction process is maximal when *d* equals zero.

Note that in the absence of experimenters (*N* = 0 in Equation (7)), no relationship emerges; the situation is the same if the experimenters are physically present, but do not pay attention to this specific experiment, or do not understand its purpose. How the relationship could become meaningful for the experimenters is described in the next section

### 3.6. The “Fossil Imprint” Hypothesis

In the first steps of the modelling, it was implicitly considered that the experimenters straightaway expected a *meaningful* relationship. Meaning supposes, however, cognitive processes and interpretation. Therefore, the question is: how did the experimenters achieve this cognitive state?

First, it is important to note that the biological systems in Benveniste’s experiments were also used for “classical” experiments. In other words, the experimenters were accustomed to study causal relationships with “genuine” molecules at micromolar concentrations: system states associated with biologically-active molecules and control conditions were compared. Moreover, these compounds at pharmacological doses were also included as positive controls in experiments aimed to evidence effects related to the “memory of water”.

Second, when bench scientists repeat identical experiments over and over, a process comparable to *classical conditioning* possibly occurs unbeknown to them. Indeed, classical pharmacological compounds or control conditions (unconditioned stimuli) are referred to their respective “labels” (conditioned stimuli). Therefore, the experimenters unthinkingly learn to associate the “labels” and the respective system states into a relationship. Note that the conditioning is reinforced by everyday experimental observations of the relationship regardless of the type of relationship, “causal” or “quantum-like”. After this learning process, the relationship becomes a new “object” that is recognized as such, in its wholeness.

Third, the relationship learned by the experimenters via classical conditioning is an abstract construct that, according to the modelling, is revealed in the macroscopic world through a quantum-like structure. The quantum-like relationship described in the modelling is similar to a reflection or an *imprint* of the initial relationship because it has some of its characteristics, but not all. Thus, even though labels and system states appear to be engaged in a relationship from the point of view of the experimenters, this link is not causal.

In the model, the “shape” of a causal relationship is thus “transferred” to a quantum-like abstract structure. The components of the causal relationship have however lost their identity, only the global form is maintained (the causal links between components have disappeared). A comparison can be made with the fossilization process where organic molecules are progressively replaced by minerals. After complete disappearance of the organic matter that composed the living organism, its *form* remains *imprinted* in stone. This form has become independent of the initial material support (even though matter is necessary to reveal it). The forces that causally linked each part of the living organism to the others have been lost and the different parts of the fossil are only juxtaposed (no local causes explain the form of the fossil).

In summary, the quantum-like correlations observed in Benveniste’s experiments could be interpreted as the “fossil imprint” of previously-observed causal relationships; this imprint is the consequence of a classical conditioning process that occurs during the repetitive experimental daily work.

## 4. Discussion

The characteristics of Benveniste’s experiments are properly described in the model: emergence of an “activated” system state, the relationship between labels and system states, and the loss of relationship in blind experiments with remote supervisor. It is, therefore, unnecessary to invoke water or something else as a support for specific information on biologically-active molecules. In other words, there is neither “memory of water” nor “ghosts of molecules”. Beyond the specific case of Benveniste’s experiments, this probabilistic modelling suggests that the conclusion of an experiment could depend on how the experimenters grasp the “reality”, either expecting a meaningful relationship or only unconnected variables (i.e., a “form” vs. separate dots). Therefore, Benveniste’s experiments could have been an instance of a more general phenomenon.

The fact that a result about a relationship is a property of the observed system and the observer taken as a whole is the basis of the modelling. In Figure 4A, due to the instability introduced in the modelling, a significant relationship emerges from random probability fluctuations. During this process where the probability of a meaningful relationship changes from 1/2 (no relationship) to 1 (certainty of a relationship), the relationship gains its existence, whereas its components lose their identity. If the experiment has been designed to ensure that the components keep their identity (i.e., with a remote supervisor), then the significant relationship vanishes and the emergent “activated” states are evenly distributed among the “labels”.

Not surprisingly, the correlations that appear between labels and system states have a quantum-like structure. Indeed, in quantum mechanics outcomes also do not pre-exist their measurements and the underlying logic of the model is comparable to Young’s two-slit experiment, an emblematic experiment of quantum physics. In Young’s experiment, if the experimenter decides to observe the phenomenon in its wholeness, light interference patterns appear on the screen. In contrast, if the experimenter decides to break down the phenomenon into elementary sub-events which are individually identified (photons passing through path “1” or path “2”), then the system adopts a corpuscular behaviour without interferences. The parameter *d* in Equation (14) could be understood as the abbreviation for “detection” of the “paths” *Pcb*_0_ and *Pcb*_1_ in Figure 5. For *d* = 0 (no detection), “interferences” are observed and for *d* = *pq* (maximal detection), the phenomenon is divided in sub-events and Equation (18) applies.

A condition for the emergence of these quantum-like interferences is the consideration of probability fluctuations. Often fluctuations are neglected in theoretical models because they have limited consequences. This is the case for example with Equation (17) for unconnected variables. In contrast, the importance of fluctuations appears in Equation (7) where they reveal that the initial states of the probability of a direct relationship are unstable; the probability then rapidly tends toward one of the two possible stable solutions at random. It is interesting to note that Aerts described macroscopic models where a quantum structure appeared as a consequence of fluctuations due to the interaction between the measurement apparatus and the system [28].

Outcomes that do not pre-exist to their measurements remind concepts from Gestalt theory [29]. According to this theory, the human mind perceives objects as a shape (Gestalt) that is independent of its parts. Amann has described well the structural similarities between Gestalt concepts and quantum mechanics:

“Similarly as with the Gestalt concept, the shape of a quantum object does not a priori exist but it depends on the interaction of this quantum object with the environment (for example: an observer or a measurement apparatus).

Quantum mechanics and Gestalt perception are organized in a holistic way. Subentities do not necessarily exist in a distinct, individual sense.

*In quantum mechanics and Gestalt perception objects have to be created by elimination of holistic correlations with the ‘rest of the world’ ”* [30].

An issue important to emphasize is the non-causal characteristics of the correlations between “labels” and system states in the model, which is a hallmark of quantum (-like) correlations. Indeed, “labels” cannot be considered as the causes of the observed system states; “labels” and states are like coincident events. As a consequence, the observed correlations cannot serve to send a message or an order; otherwise the distribution of outcomes according to supposed “causes” becomes scrambled. This is precisely what happened for the correlations between labels and states that vanished with a remote supervisor in Benveniste’s experiments.

A perspective from an uninvolved standpoint was adopted in the model. The reader must resist the temptation to put her/himself in the place of the experimenters. Indeed, as established by Breuer, a complete self-measurement is impossible [31,32]. This author demonstrated that a measurement apparatus or an observer *O* cannot distinguish all the states of a system in which it/he is contained (*O-S* in the modelling). A second external apparatus/observer (*P* in the model) is necessary to describe all the states of the first apparatus/observer. The nature of the system, classical or quantum, does not matter.

An implicit assumption of the uninvolved standpoint coupled with the intersubjective agreement is that “reality” exists only through measurements or interactions. The chosen perspective is more epistemic than ontic and the price to pay is a weakness of realism; in other words, asking on an “absolute reality” of the world outside measurements is pointless. This position is close to the Copenhagen interpretation and other interpretations of quantum physics [33,34,35]. According to these interpretations, the quantum formalism does not describe “reality” as “it is”, but describes our *relationship* with it. The consequence is the absence of an absolute standpoint. The quantum formalism allows predicting the results of measurements performed by devices considered as classical objects (these classical measurement devices being nothing more than an extension of our sensory organs). What is guaranteed in an epistemic perspective is the *consistency of the correlations* between measurements (or interactions), not their specific content.

Although the present model does not need hypotheses about water properties, the existence of “water memory” cannot be completely disproved, particularly in other experimental settings. Thus, Montagnier et al. reported experimental results that were presented as a continuation of Benveniste’s experiments [36]. More specifically, Montagnier et al. proposed that highly-diluted DNA of some bacteria and viruses could emit low-frequency electromagnetic signals. After recording, DNA information could be transmitted to water and carried in water nanostructures. Furthermore, the same authors reported that classical polymerase chain reaction could be performed by using specific DNA information stored in these nanostructures [37]. Montagnier’s hypothesis of water nanostructures is clearly in favour of a role of water as a carrier of molecular information. Therefore, it would be interesting to perform local vs. remote blind experiments in order to assess if “memory of water” is really at work in these experiments. Recently, Thieves et al. have compared local and remote blinding in a model of wheat germination with a highly-diluted compound. The results were in favour of the present model since a significant effect with high dilutions was observed only in local blind conditions [38]. As in Benveniste’s experiments, correlations between “labels” and biological states vanished in conditions of remote blinding.

Previous studies in experimental psychology have shown that cognitive processes, such as decision-making, memory, judgment, reasoning, language, or perception, could be described with mathematical quantum tools, thus offering a generalized probability theory for these processes [39]. More general than the classical approach, the present modelling also offers the possibility of new interpretations for some questions debated in different areas of biology, medicine or psychology. As an example, proponents of alternative medicines, such as homeopathy, claim that there is a relationship between their medicines and the improvement of patient symptoms [40]. However, according to evidence-based medicine, no causal relationship is evidenced in double-blind trials and opponents to these alternative treatments conclude that they are nothing more than placebos [41,42]. A new approach considering not only the “biological systems” (patients), but also the various “experimenters” and participants (physicians, patients, statisticians, etc.) could be fruitful. Similarly, studies on the placebo effect could also benefit from this original perspective if the “meaning” of the medicines—for both patients and physicians—is also considered [43,44].

Another possible application of the present approach is the current debate about reproducibility in biology, medicine, oncology, and psychology [45,46,47,48]. Of course, classical explanations are probably involved in most cases that are questioned in this “replication crisis” [49]. We have seen how the emergence of apparent causal relationships could vary according to experimenters’ characteristics (e.g., experimenters’ commitment, meaning of outcomes, training for a specific experiment). One cannot exclude that an experimenter effect related to the “imprint” hypothesis is at work for some teams but not for others when poor reproducibility among teams is reported.

## 5. Conclusions

The experimenter effect described in this model meets all requirements to explain Benveniste’s experiments, including their unusual features. No hypotheses about water properties or other exotic explanations are needed. This model could be extended to other experimental situations in biology, medicine, and psychology.

## Figures and Tables

**Table 1:** The antagonistic pro and con arguments for molecular-like effects without molecules (“memory of water” ^a^ or “digital biology” ^a^) in Benveniste’s experiments.

*Pro* Arguments	*Con* Arguments
Emergence of an “activated” state of the experimental system Apparent causal relationship between “inactive”/“active” samples and “resting”/”activated” states of the experimental systems Numerous consistent results Success of blind experiments with local supervisor ^b^	Not compatible with the physics and chemistry of liquid water Absence of a theoretical framework Reproduction of experiments by other teams generally failed Failure of blind experiments with remote supervisor ^b^

^a^ “Memory of water” is the hypothesis that specific biological information could be still present (whatever its form) in water samples after the biologically-active molecules have been removed (via extensive dilutions) or if the “activity” of these molecules has been “transmitted” via various devices (“electronic transmission” and “digital biology”). ^b^ Blind experiments with local or remote supervisor (see text).
